# The interaction of the bioinsecticide PA1b (Pea Albumin 1 subunit b) with the insect V-ATPase triggers apoptosis

**DOI:** 10.1038/s41598-017-05315-y

**Published:** 2017-07-07

**Authors:** Vanessa Eyraud, Séverine Balmand, Lamis Karaki, Isabelle Rahioui, Catherine Sivignon, Agnès F. Delmas, Corinne Royer, Yvan Rahbé, Pedro Da Silva, Frédéric Gressent

**Affiliations:** 1 Univ Lyon, INSA-Lyon, INRA, BF2I, UMR0203, F-69621 Villeurbanne, France; 2 Centre de Biophysique Moléculaire, CNRS UPR 4301, Rue Charles Sadron, 45071 Orléans, Cedex 2 France; 3 Univ Lyon, INRA, INSA-Lyon, CNRS UMR 5240 MAP, F-69622 Villeurbanne, France; 4 Laboratoire des Symbioses Tropicales et Méditerranéennes (LSTM), UMR IRD/SupAgro/INRA/UM2/CIRAD, F-34398 Montpellier, France

## Abstract

PA1b (Pea Albumin 1, subunit b) peptide is an entomotoxin, extracted from Legume seeds, with a lethal activity towards several insect pests, such as mosquitoes, some aphids and cereal weevils. This toxin acts by binding to the subunits c and e of the plasma membrane H^+^-ATPase (V-ATPase) in the insect midgut. In this study, two cereal weevils, the sensitive *Sitophilus oryzae* strain WAA42, the resistance *Sitophilus oryzae* strain ISOR3 and the insensitive red flour beetle *Tribolium castaneum*, were used in biochemical and histological experiments to demonstrate that a PA1b/V-ATPase interaction triggers the apoptosis mechanism, resulting in insect death. Upon intoxication with PA1b, apoptotic bodies are formed in the cells of the insect midgut. In addition, caspase-3 enzyme activity occurs in the midgut of sensitive weevils after intoxication with active PA1b, but not in the midgut of resistant weevils. These biochemical data were confirmed by immuno-histochemical detection of the caspase-3 active form in the midgut of sensitive weevils. Immuno-labelling experiments also revealed that the caspase-3 active form and V-ATPase are close-localized in the insect midgut. The results concerning this unique peptidic V-ATPase inhibitor pave the way for the utilization of PA1b as a promising, more selective and eco-friendly insecticide.

## Introduction

Almost one-third of world agricultural production would be lost due to pathogens or insects in the absence of chemical treatment, and even up to 80% in some areas^1^. Due to their efficiency and low cost, chemical pesticides are widely used around the world, but their extensive use leads to significant damage to ecosystems, for example by targeting different groups of beneficial insects or by impairing animal and human health^2^. Finding efficient new molecules that have less impact on the environment is a major concern in order to develop biopesticides for sustainable and healthy agriculture. From this perspective, PA1b (Pea Albumin 1, subunit b) is a promising alternative molecule. PA1b is an entomotoxin extracted from pea seeds and, more generally, from Legume seeds^3^. PA1b is active orally and it displays outstanding insecticidal activity against certain insects, such as cereal weevils (genus *Sitophilus*) and mosquitoes^4–6^. PA1b was first discovered in 1986 and consists of 37 amino acids, including six cysteines involved in three intramolecular disulfide bridges^7^. Analysis of the three-dimensional structure, determined by NMR and molecular modeling, has revealed that it belongs to the Inhibitor Cystine Knot (ICK) or knottin family^8^. The ICK family includes numerous peptides with various biological activities acting on different molecular targets^9, 10^. The insecticidal activity of PA1b has been patented^3^, and PA1b has many attributes for use on an industrial scale: it is extracted from commonly grown plants that are regularly consumed by humans and other mammals, and it is suitable for use in transgenic plants. Nevertheless, for widespread application, and particularly for use in organic farming, the complete action mechanism of the toxin needs to be described. Using an I^125^ labeled toxin, PA1b-I^125^, a unique binding site of PA1b was found on extracts from sensitive strains of cereal weevil and this binding site was undetectable in extracts from resistant strains^11^. Electrophysiological and biochemical studies revealed that PA1b targets a protein complex named vacuolar-ATPase (V-ATPase)^12^. V-ATPases are large (around 1MDa), multi-subunit complexes composed of a peripheral V1 domain that hydrolyzes ATP and an integral Vo domain that translocates protons by a rotary mechanism^13^. In insects, the V-ATPases are present on the midgut brush border and, by acidification of the intestinal lumen, they provide energy for the transport of nutrients across this membrane^14^. PA1b, the first V-ATPase peptide inhibitor, binds to the Vo domain, more specifically on subunits c and e^15^. Thus, PA1b blocks the V-ATPase activity on the insect midgut, leading to insect death. However, the precise steps, from the PA1b/V-ATPase interaction to the death of the insect, remain unknown. In mammals, the most extensively studied V-ATPase inhibitor, bafilomycin, triggers cell death by apoptosis following interaction with the V-ATPase^16–21^. In the *Drosophila melanogaster* insect model, the mechanism of apoptosis is very close to that in mammals. The key enzymes of the caspase family are well conserved, even though differences exist in the numerous pathways leading to caspase activation and apoptosis^22, 23^. A previous study showed the presence of apoptotic bodies following the PA1b intoxication of Sf9 insect cells^24^. However, nothing is known about the triggering of apoptosis after the inhibition of V-ATPase in whole insects. Three situations occur in whole insects upon Pa1b intoxication: (i) insects, such as the *Sitophilus oryzae* strain WAA42, are sensitive to the toxin and PA1b-I^125^ binds to their V-ATPase receptors; (ii) insects, such as the *Sitophilus oryzae* strain ISOR3, are totally resistant to the toxin due to the non-interaction with V-ATPase; and (iii) insects, such as the red flour beetle *Tribolium castaneum*, were found to be insensitive although the PA1b-I^125^ binding site exists on extracts from this insect, with characteristics similar to those of the sensitive insects^5, 6^. These three insect models, the sensitive *S. oryzae* strain WAA42, the resistant *S. oryzae* strain ISOR3 and the insensitive *T. castaneum*, were used in biochemical and histological experiments to demonstrate that a PA1b/V-ATPase interaction triggers the apoptosis mechanism, resulting in insect death.

## Results

### PA1b ingestion induces morphological changes in the midgut of the *S. oryzae* sensitive strain

**Figure 1:**
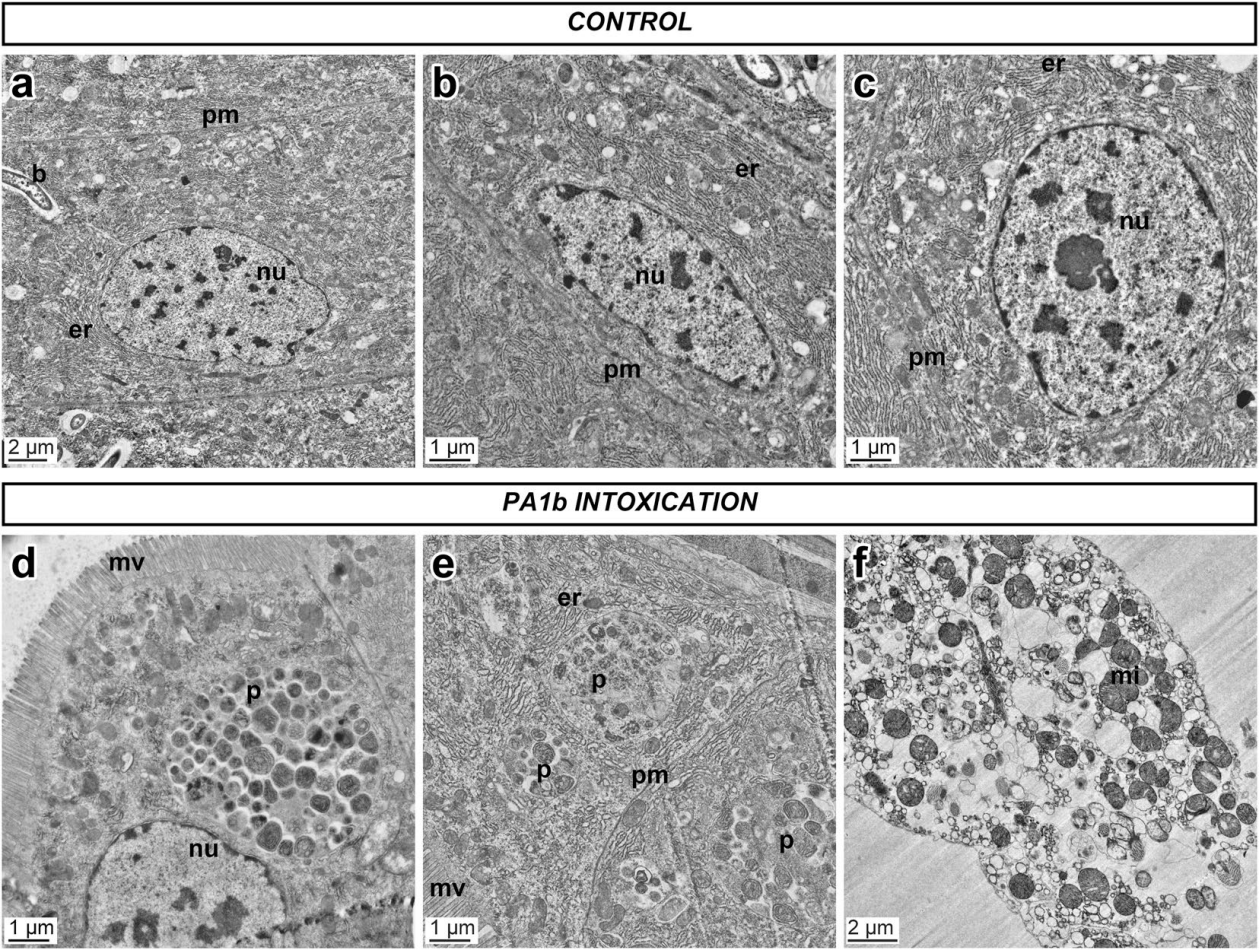
Midgut cells from a PA1b sensitive *S. oryzae* strain (WAA42) observed with transmission electron microscopy. Insect midguts were dissected, fixed, and ultra-thin sections (70 nm) were prepared as described in the Experimental Procedures section. The sensitive weevil WAA42 was fed on wheat flour either without (Control, upper panel a,b,c), or with PA1b (400 µg per g of food) for 24 h (lower panel, d,e,f). b: bacterium; er: endoplasmic reticulum; mi: mitochondria; mv: microvilli; nu: nuclei; p: phagosome; pm: plasma membrane.

The PA1b sensitive weevils, WAA42, were treated, for 24 h, with PA1b (400 µg per g of food). The midguts were collected and ultrathin sections were observed with electron microscopy. Cells of control midguts from non-intoxicated sensitive weevils had a normal appearance (Fig. 1a,b and c), with a well-defined nucleus, an abundant endoplasmic reticulum and the presence of some endosymbiotic bacteria. In contrast, the midgut cells of PA1b-treated sensitive weevils (Fig. 1d,e and f) were completely disorganized and most of them were lysed (1 f). In cells which were not lysed, the plasma membrane was hardly distinguishable, few nuclei were present and the endoplasmic reticulum was scattered. Also, phagosomes were visible in most of the cells.

### Influence of PA1b intoxication on caspase-3 activity

**Figure 2:**
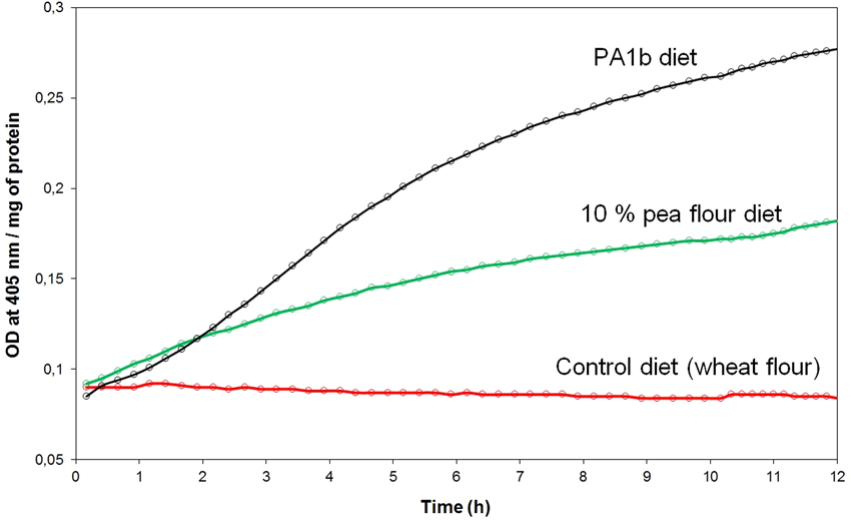
Caspase-3 activity on weevil midguts following PA1b intoxication. The weevils of the PA1b sensitive strain WAA42 were intoxicated for 24 h with an artificial diet composed of wheat flour (control, red curve); PA1b incorporated in the pea flour (10%, green curve); or PA1b (400 µg/g of food, black curve). After intoxication, the midguts were dissected and the caspase -3 activities were measured using the artificial substrate DEVD-pNA.

**Figure 3:**
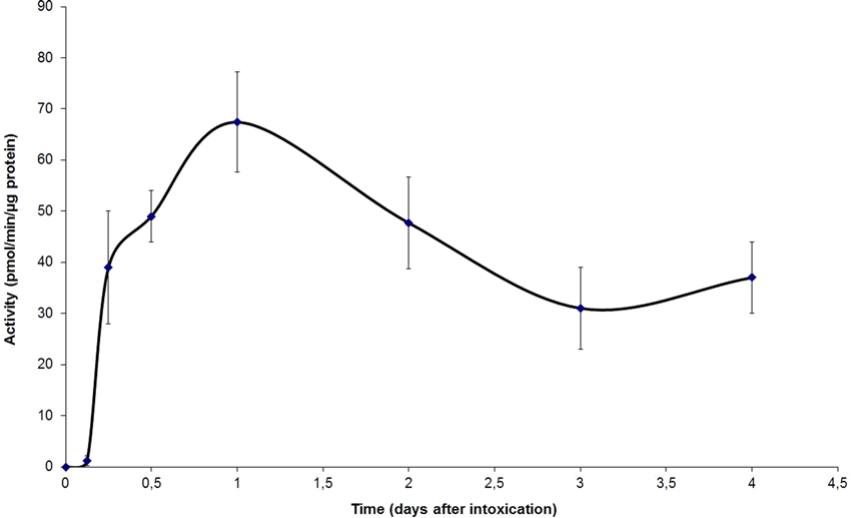
Induction kinetics of the caspase-3 activity by PA1b. The weevils of the PA1b sensitive strain WAA42 were intoxicated for different time periods (0, 3, 6, 12, 24, 48, 72 and 96 h) with an artificial diet composed of wheat flour incorporating PA1b (400 µg/g of food). After intoxication, the midguts were dissected and the caspase -3 activities were measured using the artificial substrate DEVD-pNA.

The caspase-3 activity has been revealed on midgut extracts dissected from weevils (the PA1b sensitive strain WAA42) fed, for 24 h, with pea flour (10%) or with PA1b (400 µg per g of food). The results, presented in Fig. 2, show that in weevils treated with either pea flour or PA1b, the caspase-3 activity was measured at 27.8 and 64.4 pmol/min/µg of protein, respectively. On the other hand, no detectable enzyme activity was detected in the control assay (without PA1b or pea flour in the food). Next, a kinetic assay of the caspase-3 activity was realized on weevils intoxicated for time periods ranging from 3 h to 4 days (Fig. 3). The kinetics demonstrated that the caspase-3 activity begins to be visible 6 h after exposure to PA1b and increases until it reaches a maximum at 24 h. Above this level, the activity decreases slowly until day 4 (Fig. 3). The control assays showed no detectable caspase-3 activity at any tested time. The maximum activity, at 24 h after PA1b intoxication, corresponds to a calculated activity of 67.4 +/− 9.8 pmol/min/µg of protein. Hence, the subsequent experiments were conducted with a treatment time period of 24 h.

### Specificity of the caspase-3 activity induced by PA1b

**Figure 4:**
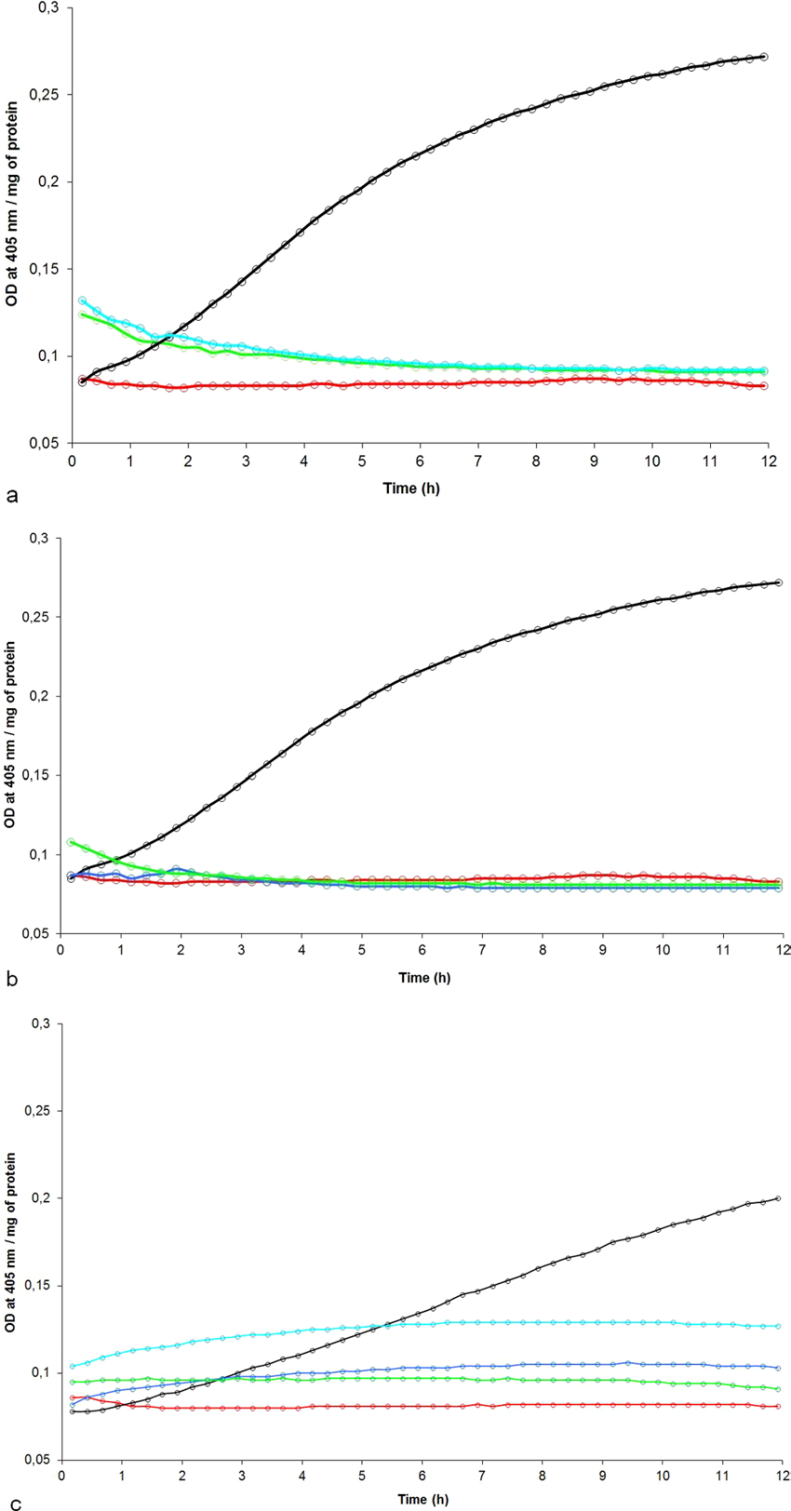
Specificity of the caspase-3 activity induced by PA1b. The weevils were intoxicated for 24 h with PA1b, 400 µg per g of food. After intoxication, the midguts were dissected and the caspase -3 activities were measured using the artificial substrate DEVD-pNA. (**a**) Sensitive weevil strain WAA42 intoxicated with PA1b (black) and control (without toxin, red); resistant weevil strain ISOR3 intoxicated with PA1b (blue) and control (green). (**b**) Sensitive weevil strain WAA42 intoxicated with PA1b (black); with reduced and alkylated PA1b (green); with the inactive mutant F10A (blue); and control (red). (**c**) Sensitive weevil strain WAA42 intoxicated with PA1b (black); control (red); with bafilomycin at 0.25 mM (dark blue) and at 0.8 mM (light blue); resistant weevil strain ISOR3 intoxicated with bafilomycin at 0.8 mM (green).

Next, the specificity of the caspase-3 activity has been tested; first by measuring the activity induced in the PA1b resistant weevil strain, ISOR3. The results presented in Fig. 4a show that, compared to the WAA42 weevil strain control assay, there was no detectable caspase-3 activity on the extract of treated ISOR3 weevil midgut. In the same way, treatment of WAA42 weevils with the inactive PA1b mutant F10A or with the reduced and alkylated peptide resulted in an absence of caspase-3 activity (Fig. 4b). The V-ATPase inhibitor bafilomycin induced caspase-3 activity in the WAA42 weevil strain: 13.8 pmol/min/µg of protein, with similar results for doses of 150 or 500 µg per g of food (respectively 0.25 and 0.8 mM) (Fig. 4c). This activity is lower than the activity induced by PA1b (24.8 pmol/min/µg of protein). However, the bafilomycin inhibitor is not able to induce caspase-3 activity in the midgut from the resistant weevil strain ISOR3, for concentrations up to 500 µg per g of food (0.8 mM).

### Effect of PA1b on caspase-3 activity in *T. castaneum*

**Figure 5:**
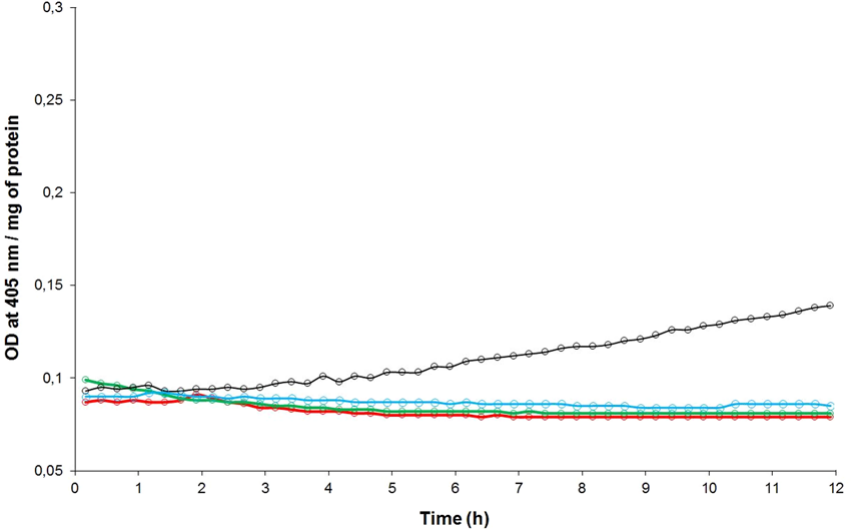
Caspase-3 activity on *T. castaneum* midguts following PA1b intoxication. The red flour beetles were intoxicated for 24 h with an artificial diet composed of 95% wheat flour and 5% yeast extract (control, blue curve). PA1b was incorporated in the diet (400 µg per g, green curve); or pea flour containing PA1b was added to the diet (10%, red curve or 25% black curve). After intoxication, the midguts were dissected and the caspase-3 activities were measured using the artificial substrate DEVD-pNA.

*T. castaneum*, a beetle which is insensitive to PA1b but which exhibits a binding site similar to the one found in weevils^5^, was treated with PA1b and the caspase-3 activity was measured. The results show that, at the concentrations used for weevils (i.e. 10% pea flour or 400 µg PA1b per g of food), there was no detectable caspase-3 activity in the midgut of *Tribolium* (Fig. 5). However, when the amount of pea flour was increased, up to 25% in the food, a slight caspase-3 activity was detectable, at a calculated level of 8.7 pmol/min/µg of protein (Fig. 5).

### Caspase-3 active form is localized in the insect midgut cells treated with PA1b

**Figure 6:**
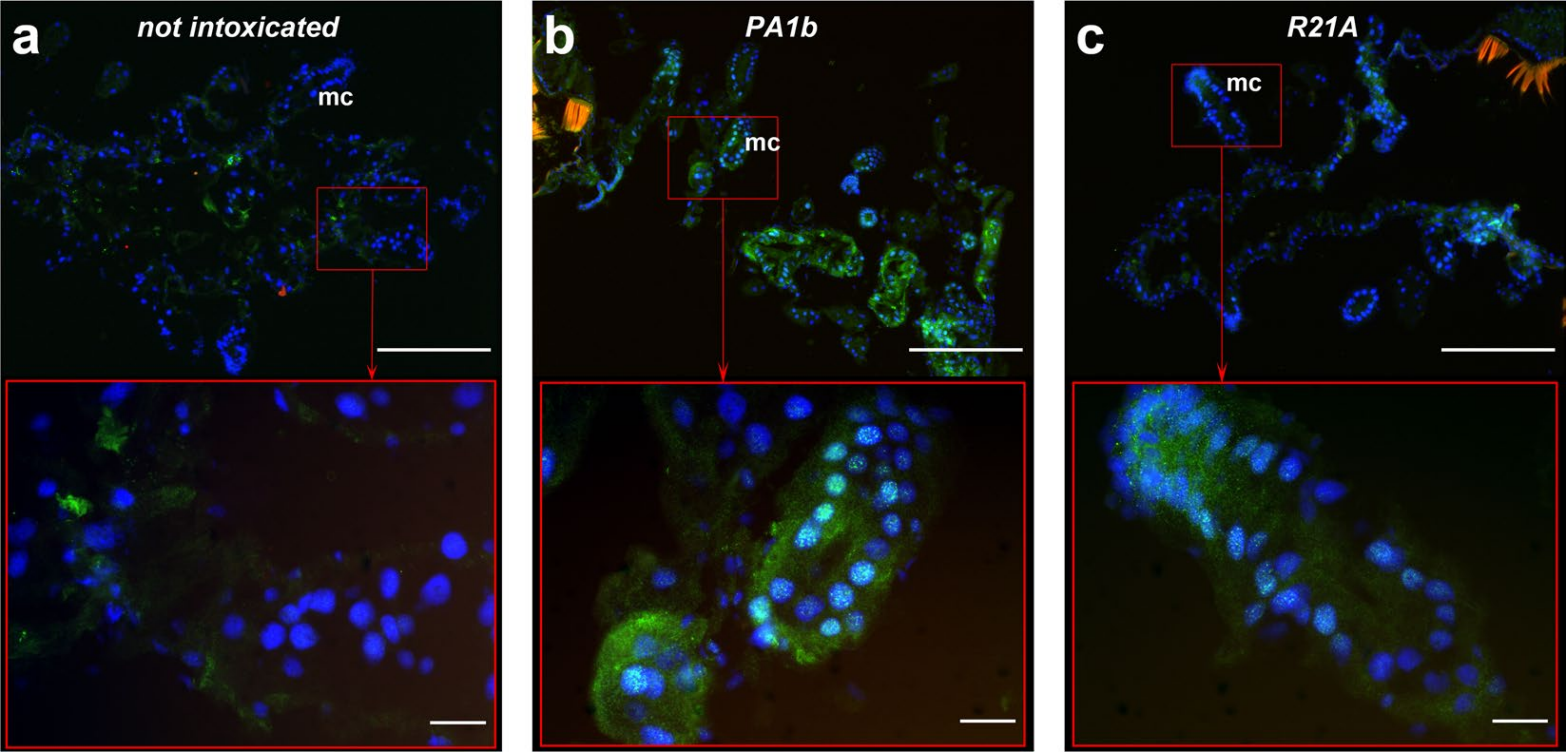
Immuno-localization of the caspase-3 active form in sensitive *S. oryzae* (strain WAA42) intoxicated by PA1b. Midguts of the sensitive weevil WAA42 were dissected, embedded, frozen and then tissue sections (7 µm) were cut. Slides were prepared as described in the Experimental Procedures section. Caspase-3 was labelled with rabbit anti-cleaved caspase-3 and revealed using a secondary antibody Alexa fluor 488 donkey anti-rabbit IgG. DAPI was added for nuclear staining. (**a**) Control (no PA1b); (**b**) weevils intoxicated with PA1b, at 400 µg per g of food, for 4 days; (**c**) weevils intoxicated with the inactive mutant of PA1b, R21A, at 400 µg per g of food, for 4 days. Upper panel scale bar = 200 µm; lower panel scale bar = 20 µm. Blue: nuclei stained with DAPI; green: caspase 3; brownish-red: non-specific signal from background autofluorescence. mc: mesenteric caeca.

**Figure 7:**
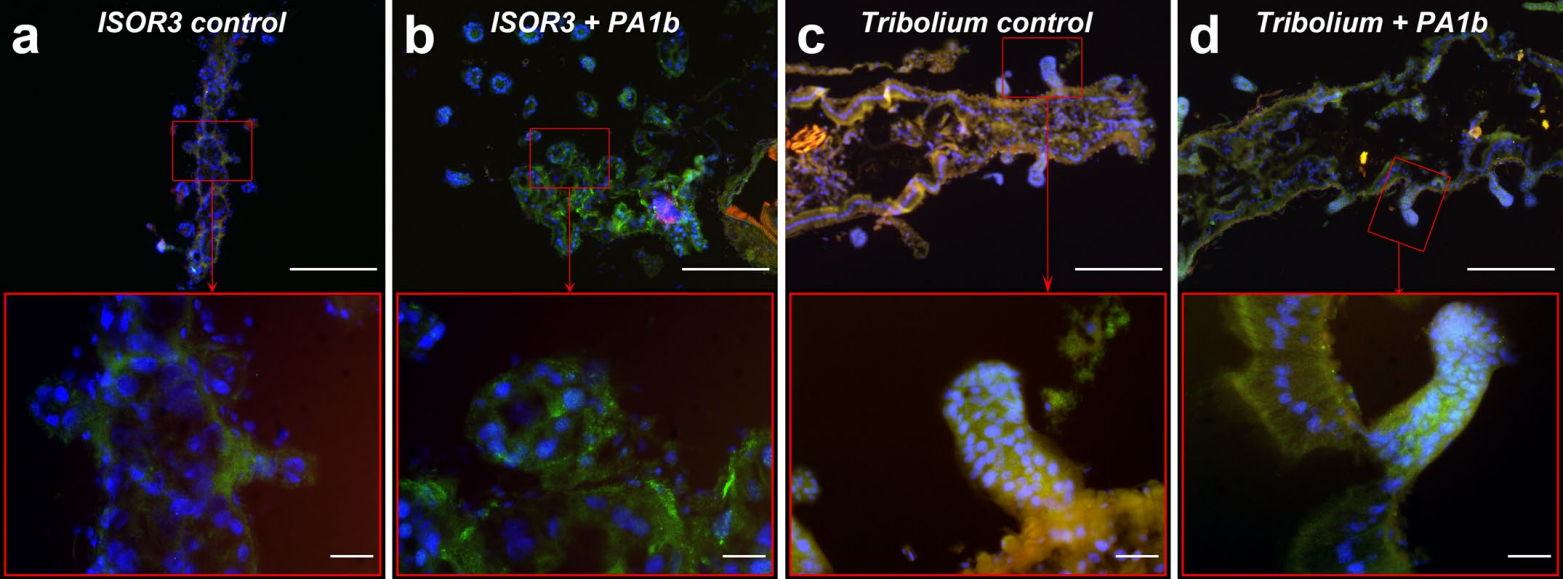
Immuno-localization of the caspase-3 active form in resistant *S. oryzae* (strain ISOR3) and in the insensitive insect *T. castaneum*, intoxicated with PA1b. Insect midguts were dissected, embedded, frozen and then tissue sections (7 µm) were cut. Slides were prepared as described in the Experimental Procedures section. Caspase-3 was labelled with rabbit anti-cleaved caspase-3 and revealed using a secondary antibody Alexa fluor 488 donkey anti-rabbit IgG. DAPI was added for nuclear staining. The midgut of the resistant weevil strain ISOR3 reared on wheat flour (control, **a**) or intoxicated with PA1b, at 400 µg per g of food, for 4 days (**b**); the midgut of the insensitive insect *T. castaneum* reared on wheat flour (control, **c**) or intoxicated with PA1b, at 400 µg per g of food, for 4 days (**d**). Upper panel scale bar = 200 µm; lower panel scale bar = 20 µm. Blue: nuclei stained with DAPI; green: caspase 3; brownish-red: non-specific signal from background autofluorescence.

All histological experiments were conducted on insects (*S. oryzae* strains WAA42 and ISOR3 and *T. castaneum*) treated for 4 days, using 400 µg per g of food for PA1b and 150 µg per g of food for bafilomycin. When the *S. oryzae* sensitive strain (WAA42) insects were reared on non-intoxicated medium (Fig. 6a), the immuno-localization of the caspase-3 active form in gut sections revealed an absence of this enzyme. When the same strain was reared with food containing PA1b, the caspase-3 active form was detected throughout the midgut and, particularly, in the mesenteric caeca (Fig. 6b). When the medium contained an inactive PA1b mutant, R21A, a very faint signal indicated the presence of the caspase-3 active form (Fig. 6c). Similarly, when resistant (*S. oryzae* ISOR3) and insensitive (*T. castaneum*) insects were reared on untreated medium (Fig. 7a and c), the caspase-3 active form was not detected in the midguts. When PA1b was added to the medium, there was a slight detection of caspase-3 in the midgut of resistant weevils and *T. castaneum* (Fig. 7b and d). Similarly, when resistant (*S. oryzae* ISOR3) and insensitive (*T. castaneum*) insects were reared on untreated medium (Fig. 7a and c), the caspase-3 active form was not detected in the midguts. When PA1b was added to the medium, caspase-3 was detected at a very low level in the midgut of *T. castaneum* (Fig. 7d), but not in resistant weevils (Fig. 7b).

### V-ATPase (receptor of PA1b) and caspase-3 are close-localized

**Figure 8:**
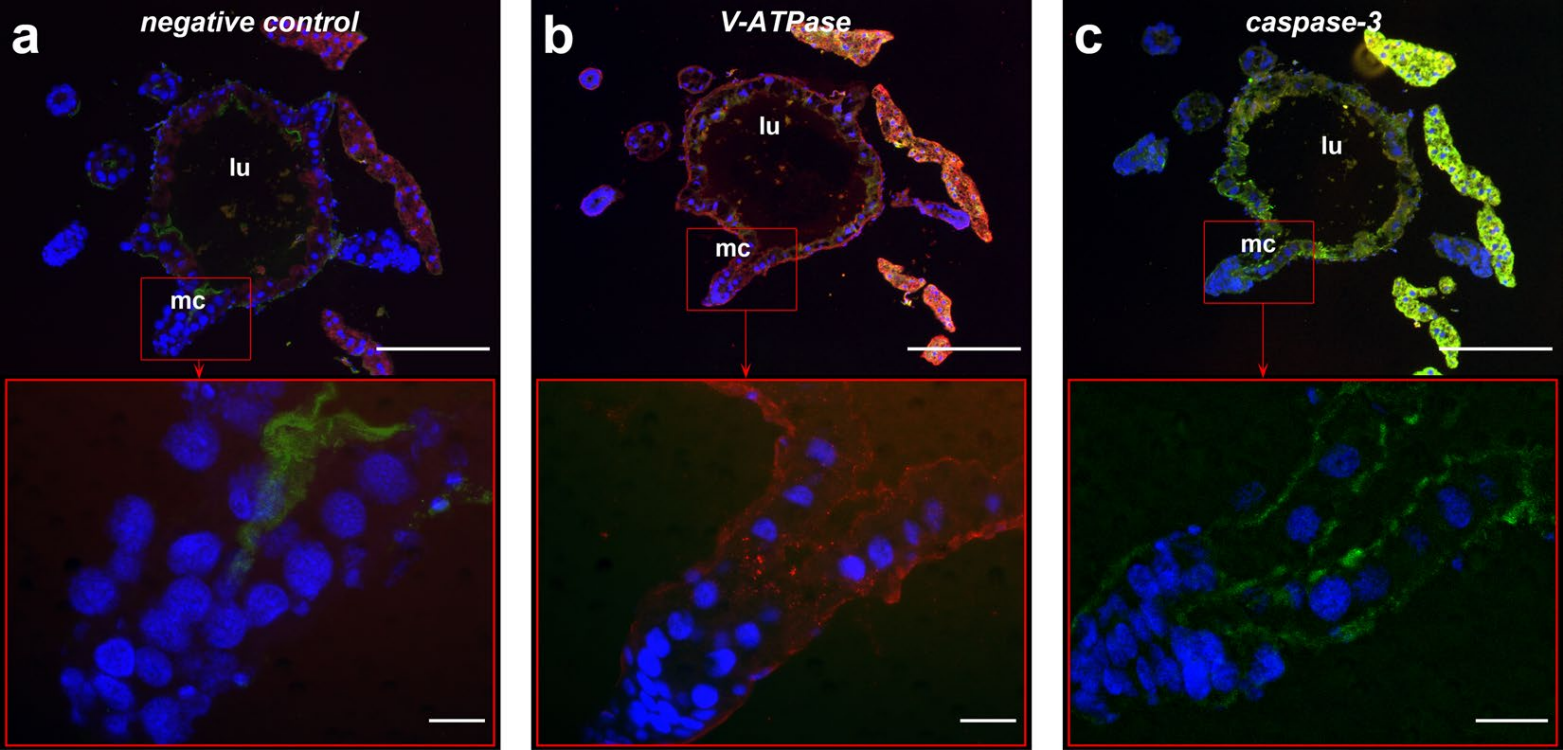
Immuno-localization of the caspase-3 active form and V-ATPase in sensitive *S. oryzae* (strain WAA42) intoxicated with PA1b. Midguts of the sensitive weevil WAA42 were dissected, embedded, frozen and then tissue sections (7 µm) were cut. Slides were prepared as described in the Experimental Procedures section. Caspase-3 was labelled with rabbit anti-cleaved caspase-3 and V-ATPase was labelled with a rabbit anti-ductin antibody. Revelation of both antibodies was performed using a secondary antibody Alexa fluor 488 donkey anti-rabbit IgG. To compare the labelling of caspase-3 and V-ATPase, labelling was performed on successive cuts and the green signal of anti-V-ATPase was transformed into a red signal. DAPI was added for nuclear staining. (**a**) negative control (non-immune serum); (**b**) anti-V-ATPase antibody labelling and (**c**) anti-caspase-3 antibody labelling. Upper panel scale bar = 200 µm; lower panel scale bar = 20 µm. Blue: nuclei stained with DAPI; green: caspase 3; red: V-ATPase.

Due to technical constraints, the immuno-localization of caspase-3 and of V-ATPase was performed on three successive sections of gut from the *S. oryzae* strain WAA42, separated by 7 µm. In the first photograph (Fig. 8a), non-immune serum was applied to the slides as a negative control. Therefore, only the nuclei were stained blue and it was possible to identify the mesenteric caeca in the weevil gut. In the second section of the same gut structure (Fig. 8b), the V-ATPase was red and was localized on the caeca membrane; in the third section (Fig. 8c) the caspase-3 active form was green and was clearly localized on the same membrane as caspase-3.

### PA1b insect resistance induces bafilomycin resistance

**Figure 9:**
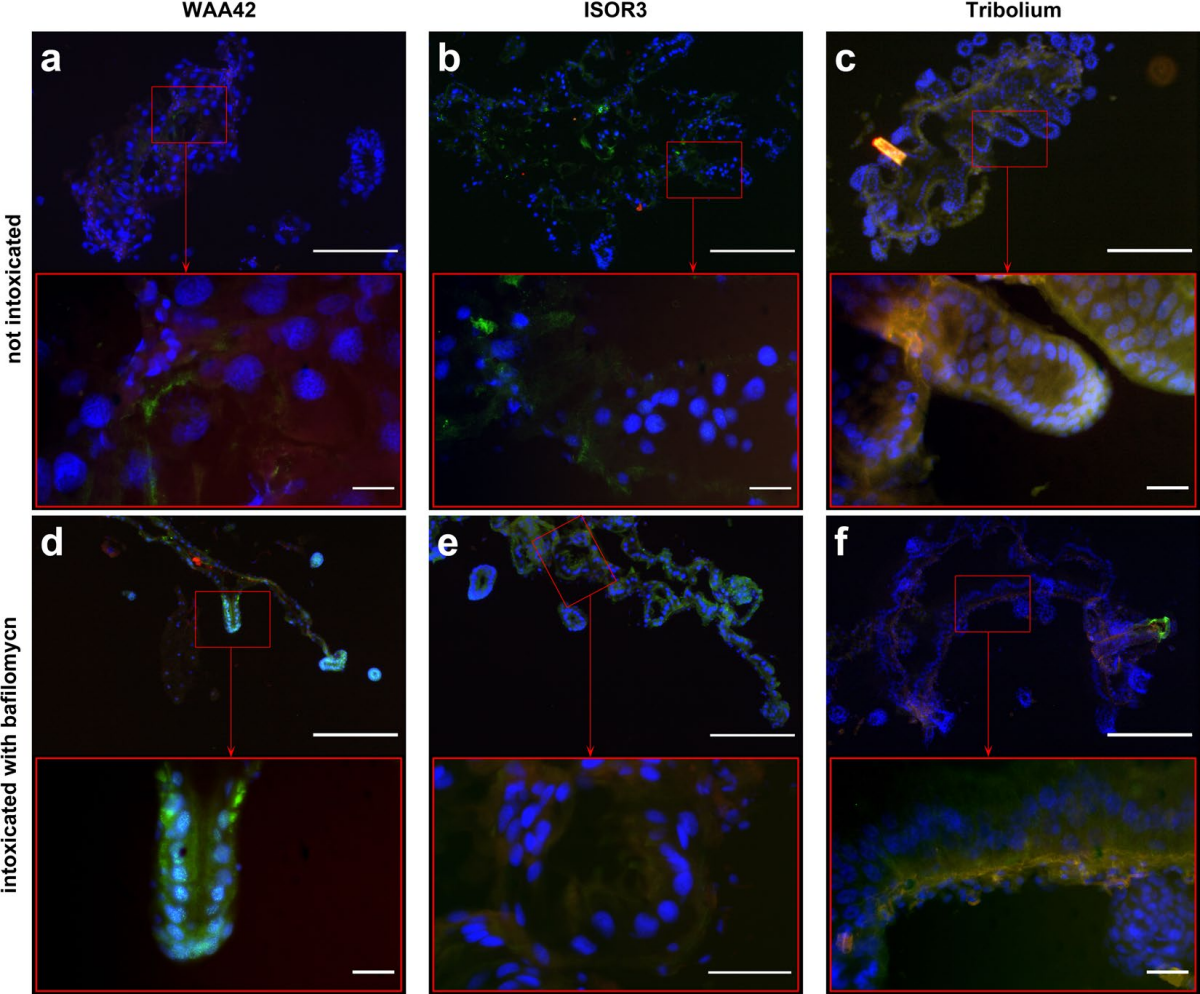
Immuno-localization of the caspase-3 active form in sensitive *S. oryzae* (strain WAA42), in resistant *S. oryzae* (ISOR3), and in insensitive *T. castaneum* intoxicated with bafilomycin. Midguts of insects were dissected, embedded, frozen and then tissue sections (7 µm) were cut. Slides were prepared as described in the Experimental Procedures section. Caspase-3 was labelled with rabbit anti-cleaved caspase-3, and revelation was performed using a secondary antibody Alexa fluor 488 donkey anti-rabbit IgG. DAPI was added for nuclear staining. The *S. oryzae* PA1b sensitive strain (WAA42), the resistant (ISOR3) strain, and the insensitive insect *T. castaneum* were all reared on non-intoxicated medium (panel a,b and c, respectively) or on the same medium containing bafilomycin (150 µg per g of food) (panel d, e, and f, respectively). Upper panel scale bar = 200 µm; lower panel scale bar = 20 µm. Blue: nuclei stained with DAPI; green: caspase 3; brownish-red: non-specific signal from background autofluorescence.

*S. oryzae* PA1b sensitive (WAA42) and resistant (ISOR3) strains, and the insensitive insect *T. castaneum*, were reared on non-treated medium (Fig. 9a,b and c) or on medium containing bafilomycin (150 µg per g of food) (Fig. 9d,e and f). After 4 days, the caspase-3 active form was detected, by immuno-localization, in midgut sections. Like PA1b, bafilomycin induced caspase-3 activity in the sensitive strain (Fig. 9d) but not in resistant weevils (Fig. 9e) or in the insensitive insect *T. castaneum* (Fig. 9f).

## Discussion

Pa1b is an insecticidal peptide extracted from garden peas and it is potentially an outstanding tool for pest control. Its receptor is the vacuolar ATPase (V-ATPase)^12^, a 1MDa transmembrane proton pump that operates *via* a rotary mechanism fuelled by ATP. In this multi-protein complex, PA1b binds to subunits e and c from the Vo complex^15^. The central role of V-ATPase in insect gut physiology^25^ means that the enzyme is also valuable as a novel potential pesticide target^26^. PA1b is the first-discovered peptidic V-ATPase inhibitor and it is, therefore, remarkable, not only because of its V-ATPase specificity and potency but also because it is uniquely selective against insects^4, 5, 24^. To date, the precise mechanism leading to insect death has remained a mystery. The experiments presented in this study used the sensitive *S. oryzae* strain WAA42, the resistant *S. oryzae* strain ISOR3, and the insensitive beetle *T. castaneum* as model insects to provide a much clearer picture of each step of the mechanism, from PA1b perception to insect death. Previous work has shown that PA1b intoxication of Sf9 insect cells induces the formation of apoptotic bodies^24^. In mammals, the specific V-ATPase inhibitors bafilomycin and apicularen also trigger apoptosis^16, 20^.

Using electron microscopy, investigations were first conducted to assess whether ingestion of PA1b had an effect on intestinal cells in the susceptible insect S. oryzae strain WAA42. Comparison of the morphological changes between the poisoned or control insect guts shows that PA1b ingestion induces many morphological changes in cells that are typical of apoptosis^27–29^. The presence of phagosomes shows that cells undergoing apoptosis are attached to phagocytes and, subsequently, degraded inside the phagosomes^30^. There are two pathways that lead to apoptosis: the membrane pathway and the mitochondrial pathway, both inducing caspase-3 activation. This enzyme is present in all cells, but in an inactive form. Only apoptosis resulted in the activation of this enzyme^31, 32^, i.e. the presence of the caspase-3 active form is a clear indicator of apoptosis. Biochemical and immuno-labelling experiments have shown the activity of caspase-3 in the PA1b-treated gut of the sensitive weevil WAA42, while there was no activity in gut tissue from non-intoxicated insects. The negative control PA1b mutants, R21A and F10A, differ from the PA1b sequence by one amino acid and this affects the ability to bind with the receptor^33^ and, our results showed here that they are unable to activate caspase-3. Furthermore, PA1b intoxication of the resistant *S. oryzae* strain ISOR3 has not led to any caspase-3 activity in the extracted insect gut. Here, the biochemical and immuno-localization techniques correlated with previous data showing no detectable binding to PA1b-I^125^ on membrane protein extracts from ISOR3^11^. All these results clearly demonstrate that the active PA1b isoform is able to bind to its receptor and induce caspase-3 activity in the sensitive weevil strain. The conclusion of our study would thus be that PA1b ingestion triggers the apoptosis of cells in insect guts, and that apoptosis induction is the result of binding of the toxin to the V-ATPase receptor.

The experiments involving immuno-localization of V-ATPase and the active caspase-3 enzyme in successive gut sections, from the *S. oryzae* strain WAA42, both showed labelling on the membrane of the mesenteric caeca. This result suggests that the V-ATPase and the caspase-3 activity are close-localized and, therefore, apoptotic activity occurs where V-ATPase is present. Upon binding to its receptor, PA1b inhibits the proton pump activity of V-ATPase, thus generating stress in the cell which leads to the induction of apoptosis and insect death. This data reinforces the conclusion that the binding of PA1b to the insect V-ATPase is required to trigger apoptosis.

The active form of caspase-3 in the insensitive insect of *T. castaneum* was investigated. Slight caspase-3 activity was detected only at very high doses of PA1b, and this activity was absent in the control insect midguts. Thus, in the insensitive *T. castaneum*, it would appear that a molecular mechanism of PA1b toxin sensitivity is present, i.e. the V-ATPase could bind to PA1b and this interaction may trigger apoptosis. This conclusion is supported by the results of PA1b toxicity tests realized on Sf9 cells^24^ from the caterpillar *Spodoptera frugiperda*. The Sf9 cells were found to be sensitive to PA1b, but living whole insects appeared to be insensitive to the peptide^6^. Considering that the affinity for V-ATPase is quite similar in sensitive and insensitive insect species^5, 11^, the insensitivity must be due to an, as yet, unidentified mechanism, and one which is very different from the mechanism of “true” resistance observed in resistant weevil ISOR3 due to an absence of binding between the PA1b toxin and the V-ATPase.

To elucidate further the initiation of apoptosis after PA1b binding to its receptor, its action with that of bafilomycin, the known inhibitor of V-ATPase was compared. Like PA1b, bafilomycin induces apoptosis in intoxicated cells^20^ and is known to act on all kinds of V-ATPase^34, 35^. However, a high concentration of bafilomycin did not induce caspase-3 activity, or any subsequent apoptosis, in *S. oryzae* ISOR3 or *T. castaneum* whereas it did induce apoptosis in *S. oryzae* WAA42 intoxicated cells (Fig. 9). These results are consistent with those obtained by Muench *et al*.^15^, showing that the *S. oryzae* ISOR3 strain was insensitive to bafilomycin (600 µg per g of food) while the WAA42 strain was sensitive at the same concentration. These two strains are quasi-isogenic lines, ISOR3 being selected on the basis of a PA1b resistance trait from WAA42 and the resistant strain China^36^. It is likely that the difference responsible for PA1b resistance induces the resistance to bafilomycin in strain ISOR3, i.e. the mutation on V-ATPase induces resistance to both PA1b and bafilomycin. This conclusion is supported by the fact that PA1b and bafilomycin share the V-ATPase subunit c as a receptor site^15^.

The PA1b peptide is a promising pesticide and, being extracted from common plants, such as soybeans and peas, it is potentially applicable in organic farming^4^. Since its host range is limited to insects^6^, it can be considered safe for mammals and other non-target organisms which may be in contact with PA1b during its use as a pesticide. However, before applying this new plant pesticide on an industrial scale, it is important to understand its mechanism of action. Together with the identification of its receptor, our present results show that the binding of this toxin to its receptor triggers an apoptotic mechanism which leads to the death of the target cells and, ultimately, the death of the insect.

Further analysis of our work may result in a new selective class of insecticides with PA1b as the lead compound in the control of insect pests, including some of the most damaging pests in global agriculture.

## Experimental Procedures

### Chemicals

One batch of the purified toxin isoform PA1b was used, with a molecular mass of 3741 Da^11^. The F10A and R21A mutants were obtained by chemical synthesis and *in vitro* oxidative folding, according to the method of Da Silva and coworkers^33^. The bafilomycin was obtained from SIGMA (Ref. 11707). The caspase-3 substrate DEVD-pNA was from Calbiochem (Ref. 235400). The monoclonal anti-Caspase-3 antibody (rabbit Cleaved caspase-3 (Asp 175) Antibody) came from Ozyme (Ref 9661). Rabbit anti-ductin polyclonal antibodies were from Chemicon International (Ref. ABS469). The anti-caspase antibody and anti-ductin antibodies were detected using fluorescent donkey anti-rabbit IgG, labelled with Alexa fluor 488 (Molecular Probes, Reference A21206, Life Technologies Ltd, UK).

### Insect rearing and intoxication

The rice weevils (*Sitophilus oryzae*, Coleoptera), sensitive strain WAA42, were reared on wheat seeds at 27.5 °C and 70% RH. The intoxications were performed for 24 h on adults feeding on food pellets (composed of wheat flour and water) incorporating the tested compound, as described in detail in^37^. The PA1b resistant strain ISOR3 was reared identically except that insects were fed on pea seeds rather than on wheat seeds. Prior to intoxication, the resistant weevils were reared for 5 days on the same wheat diet as for the sensitive strain.

The red flour beetle (*Tribolium castaneum*) was reared in the same conditions, on a diet composed of 95% wheat flour and 5% yeast extract. The intoxications were performed by incorporating the tested compound into this diet.

### Determination of the enzymatic activity of caspase-3

The caspase-3 activity was determined using the artificial substrate DEVD-pNA, according to a previously described method^38, 39^. Briefly, 10 digestive tracts (DTs) of weevils (15 for *T. castaneum*) were dissected, and 50 µL of Lysis Buffer (20 mM PIPES pH 7.2, 100 mM NaCl, 1 mM EDTA, 63 mM MgCl_2_, 10% saccharose, 0.1% CHAPS, 0.5% Nonidet P-40, 0.5% Tween 20) was added. The DTs were homogenized using a Potter, then sonicated in a bath for 10 min and centrifuged at 10 000 g for 10 min. The supernatant was collected and used immediately. The reaction was performed by adding 20 µL of DT extracts to 180 µL of reaction buffer (20 mM PIPES pH 7.2, 100 mM NaCl, 1 mM EDTA, 63 mM MgCl_2_, 10% saccharose, 0.1% CHAPS, 10 mM DTT, 0.1 mM DEVD-pNA) in a 96-well plate. The kinetics of the caspase-3 activity was monitored at 405 nm, every 15 min for 12 hours, using a PowerWave XS plate reader (BioTek). The caspase-3 activity was calculated according to the DEVD-pNa manufacturer’s method.

### Protein determination

Protein content was measured using the bicinchoninic acid procedure developed by PIERCE (Rockford, USA), with BSA as the reference.

### Transmission electron microscopy

Insect midguts were dissected and then fixed with 3% glutaraldehyde in 0.1 M Na-cacodylate buffer, pH 7.4, for 24 h at 4 °C. Midguts were stored in 0.2 M Na-cacodylate buffer, pH 7.4, until post-fixation in 1% osmium tetroxide. They were then contrasted in 1% uranyl acetate, dehydrated through an ethanol series and embedded in epoxy resin (Epon). Ultra-thin sections (70 nm) were prepared using a REICHERT Ultracut S ultramicrotome (Leica microsytems) and stained with lead citrate before examination with a Philips CM120 electron microscope. Afterwards, the contrast of the images was optimized using the PlugIn “Enhance Local Contrast (CLAHE)” on ImageJ software^40^.

### Immuno-localization of the caspase-3 and of the V-ATPase proteins

For the immuno-histochemical detection of caspase-3, insect midguts were sampled in dissection buffer (25 mM KCl, 10 mM MgCl_2_, 250 mM sucrose, 35 mM Tris-HCl pH 7.5). Then, these midguts were impregnated in a 15% saccharose, 5% glycerol, PBS buffer, pH 7.2 solution. Midguts were embedded in a gel Tissues-tek O.C.T (Sakura Finetek US) and frozen using liquid nitrogen vapour. Tissue sections, 7 µm thick, were cut using a cryostat (Microm HM 560; Thermo scientific) and the sections were placed on polylysine coated slides. The slides were then dried on a hotplate and fixed by incubation in a Finefix solution (28% Finefix (Milestone), 72% ethanol). The samples were rehydrated and washed in PBS. For caspase-3 immunostaining, unmasking of the antigenic site was performed by immersing slides in Antigen Retrieval Buffer (10 mM Tris-HCl, 1 mM EDTA, 0.05% Tween-20) for 20 min, at 99 °C. After cooling, the slides were incubated with 1% BSA in PBS for 30 min prior to an overnight incubation, at 4 °C, with rabbit anti-cleaved caspase-3 1:200 in 0.1% BSA in PBS (Cell Signaling Technology). For V-ATPase immunostaining, sections were permeabilized with triton X-100 0.1% for 10 minutes, then with sodium dodecyl sulfate 0.5% for 5 minutes. After PBS washing, the slides were incubated with 1% BSA in PBS for 30 min prior to an overnight incubation, at 4 °C, using a rabbit anti-ductin antibody 1:300 in PBS with 0.1% BSA (Chemicon International). For immunostaining negative controls, non-immune rabbit serum, diluted at 1:200 in 0.1% BSA in PBS, was used. After an initial incubation, all the sections were washed with PBS containing 0.2% Tween. The secondary antibody Alexa fluor 488 donkey anti-rabbit IgG (Molecular Probes) was applied for 1 hour, at room temperature, diluted 1:600 in PBS with 0.1% BSA. Afterwards, sections were washed with PBS-Tween, rinsed with PBS and then with several changes of water. The sections were left to dry on the bench, and mounted using PermaFluor™ Aqueous Mounting Medium (ThermoFisher Scientific, Cheshire, UK) with the addition of 4,6-diamidino-2-phenylindole (DAPI) for nuclear staining (3 µg per ml of medium). Sections were observed under epifluorescence using an IX81 Olympus microscope, with specific emission filters: HQ535/50 for a green signal, D470/40 for a blue signal (DAPI) and HQ610/75 for a red signal. Images were captured and modified using an F-View II camera coupled with Cell F software (Olympus SIS GmbH, Münster, Germany). For a comparison of caspase-3 and V-ATPase, labelling was performed on successive sections and, for clarification, the green signal of anti-V-ATPase was transformed into a red signal.
